# Finally neutralizing the threat? A novel SARS-CoV-2 vaccine platform that elicits enhanced neutralizing antibody responses

**DOI:** 10.1128/mbio.00067-24

**Published:** 2024-02-26

**Authors:** Daniel J. Salamango

**Affiliations:** 1 Department of Microbiology, Immunology, and Molecular Genetics, University of Texas Health Science Center, San Antonio, Texas, USA; Fondazione Biotecnopolo di Siena, Siena, Italy

**Keywords:** humoral immunity, pandemic, SARS-CoV-2, neutralizing antibodies, vaccines

## Abstract

The severe acute respiratory syndrome coronavirus-2 (SARS-CoV-2) outbreak took the world by storm due to its rapid global spread and unpredictable disease outcomes. The extraordinary ascension of SARS-CoV-2 to pandemic status motivated a world-wide effort to rapidly develop vaccines that could effectively suppress virus spread and mitigate severe disease. These efforts culminated in the development and deployment of several highly effective vaccines that were heralded as the beginning-of-the-end of the pandemic. However, these successes were short lived due to the unexpected and continuous emergence of more transmissible and immune-evasive SARS-CoV-2 variants. Thus, attention has shifted toward developing novel vaccine platforms that elicit more robust and sustained neutralizing antibody responses. Recent findings by Muñoz-Alía and colleagues address this by combining a live recombinant measles vaccine platform with novel biochemical approaches to generate vaccine candidates that bolster the potency of neutralizing antibody responses against diverse SARS-CoV-2 spike proteins (M. Á. Muñoz-Alía, R. A. Nace, B. Balakrishnan, L. Zhang, et al., mBio 9:e02928-23, 2024, https://doi.org/10.1128/mbio.02928-23).

## COMMENTARY

The pandemic potential of coronaviruses has been smoldering in the background since the appearance of severe acute respiratory syndrome coronavirus-1 (SARS-CoV-1) in 2003. While this outbreak was constrained to only ~10,000 cases and ~1,000 deaths, the suddenness of its emergence coupled to its transmissibility raised concerns that a future genetic recombination event could give rise to a more catastrophic problem (1). Roughly 10 years later, those fears were partially realized as the MERS (Middle East respiratory syndrome virus) coronavirus emerged in the Arabian Peninsula; luckily, this outbreak was restrained to an abortive epidemic as the virus hadn’t adapted to interhuman transmission (2). Unfortunately, that luck would run out with severe acute respiratory syndrome coronavirus-2 (SARS-CoV-2), which has caused over 770 million reported infections and roughly 7 million reported deaths and triggered the greatest global economic recession in half a century (3, 4).

The surge in global SARS-CoV-2 cases at the beginning of 2020 spurred an urgent need to develop therapeutic countermeasures that could curb virus spread and severe disease. As is the case for many viral pathogens, vaccination is the most effective therapeutic strategy to decrease morbidity and mortality. In general, viral glycoproteins that decorate the surface of enveloped viruses serve as excellent targets for host humoral responses because they’re accessible to neutralizing antibodies (nAbs) and are essential for virus replication (5–7). However, past viral outbreaks have demonstrated that this can be a double-edged sword as viral glycoproteins often accumulate mutations that alter antigenicity, rendering pre-existing immunity obsolete (8–10). This would inevitably foreshadow the continuous emergence of SARS-CoV-2 variants harboring mutations in the spike glycoprotein that permit increased transmissibility and escape from antibody recognition, leaving researchers firmly entrenched in a molecular arms-race to develop novel vaccination strategies that could potentially end the pandemic.

SARS-CoV-2 spike glycoproteins form homotrimers comprised of several key domains essential for function, including a receptor binding domain (RBD), a cell-to-cell membrane fusion peptide, and a transmembrane stalk (11, 12). Spike initiates infection by engaging the ACE2 cell surface protein through its receptor binding domain, which triggers conformational rearrangements and virus-to-cell membrane fusion to deliver the viral payload into the host cytoplasm. Several first-generation vaccines employed a genetically engineered spike protein locked in a prefusion-stabilized conformation to generate potent neutralizing IgM and IgG antibody responses (10, 13–15). Serological studies indicate that roughly 90% of these nAbs recognize the RBD and affect spike function by directly blocking ACE2 binding or limiting conformational changes required for function (10, 16, 17).

Initially, natural immunity coupled to wide-spread vaccination seemed like a promising combination for quelling the pandemic, especially considering the virus exhibited limited adaptive evolution and phenotypic changes during the early months of the outbreak (18, 19). However, over time, that changed dramatically as heavily mutated spike variants emerged, giving rise to multiple lineages classified as “variants of concern” on the basis that they exhibit substantially altered transmissibility or immune escape from vaccine-derived antibodies (20–27). Furthermore, mounting evidence has demonstrated that vaccine-induced antibody titers decrease rapidly and significantly over time, which limits their ability to block new infections and alleviate symptomatic disease, requiring booster vaccinations to restore protection (15, 28–30).

The limited duration of protective immunity provided by first-generation vaccines has motivated the development of alternative strategies that repurpose preexisting vaccine platforms or revisit vaccine designs to enhance immunologic responses to novel spike variants. This includes heterologous booster combinations, generating chimeric immunogens between different variants, or simply brute-forced production of individual vaccines for each variant (31–34). While these strategies have shown promise in driving robust nAb responses against diverse spike proteins, the major barrier of extending nAb longevity remains. An attractive solution has emerged that involves repurposing the measles vaccine to deliver SARS-CoV-2 antigens (35–37). This is particularly appealing because measles-protective nAbs are lifelong, and the vaccine has a long-standing track record of safety, which has been a major point of concern surrounding mRNA-based vaccines (38–42). However, this approach has come up short clinically with the most recent blow being a failed phase-I clinical trial due to low seroconversion rates, likely due to preexisting anti-measles antibodies (43).

A recent study in *mBio* by Muñoz-Alía and colleagues sought to address this problem by combining biochemically enhanced SARS-CoV-2 spike immunogens with a surface-modified measles-vectored vaccine (44). As mentioned above, first-generation vaccines leverage prefusion-stabilized spike monomers as antigens. However, evidence suggests that neutralizing antibodies more efficiently engage quaternary epitopes rather than their monomeric counterparts (45–47). Thus, Muñoz-Alía et al. generated vectors that express self-trimerizing full-length spike antigens in combination with additional biochemical alterations that promote more favorable spike conformations and permit the multivalent display of assembled spike trimers on the vaccine surface (48–50). Indeed, these modifications triggered substantially enhanced Th1-dominant T-cell and nAb responses in animals vaccinated with either recombinant proteins or measles-based derivatives compared with vaccination using unmodified controls. Importantly, when this strategy was expanded to include the Omicron variant, which is the predominant strain currently in circulation, Muñoz-Alía et al. observed increased antibody titers against both Omicron and the original Wuhan-1 isolate, suggesting this platform could provide protection against both heterologous and historical variants. Additionally, transfer of convalescent sera from vaccinated to naïve animals conferred robust protection against challenge with the Omicron variant.

Another key design feature implemented by Muñoz-Alía et al. was the elimination of surface epitopes that could blunt vaccine efficacy through measles immunity, which has been a barrier for this vaccine platform clinically (43, 51). This possibility was tested by inoculating animals with measles virus-specific IgG antibodies prior to immunization with “wild-type” or surface-mutated measles-vectored SARS-CoV-2 vaccines. As anticipated, the mutated measles vaccine generated robust nAb titers against SARS-CoV-2 spike antigens while the “wild-type” vaccine had no detectable nAb responses. In addition, animals vaccinated with the “wild-type” measles-vectored SARS-CoV-2 vaccine generated significant levels of anti-measles nAbs whereas the mutant vaccine had none.

This study by Muñoz-Alía et al. indicates that spike antigen optimization is equally as important as the delivery system for developing novel spike-based vaccine platforms (44). Further development and rigorous testing of this design are warranted as it may provide the key to unlocking sustainable nAb protection against current and future SARS-CoV-2 variants. More importantly, leveraging a measles-vectored vaccine could alleviate the general public’s hesitancy toward mRNA vaccines and potentially eliminate the need for semi-regular booster vaccinations, both of which could be instrumental in enticing more individuals to get vaccinated and to finally end the pandemic.
